# Supplementary material for: Drug repurposing for aging research using model organisms
Source: Aging Cell. 2017 Jun 16;16(5):1006–15. doi: 10.1111/acel.12626 (PMC5595691; doi:10.1111/acel.12626)
Supplement: Supplementary file 7 — Data S1 Zip‐Archive of all report cards. [file ACEL-16-1006-s007.zip › RC_IZG.pdf]

IZG

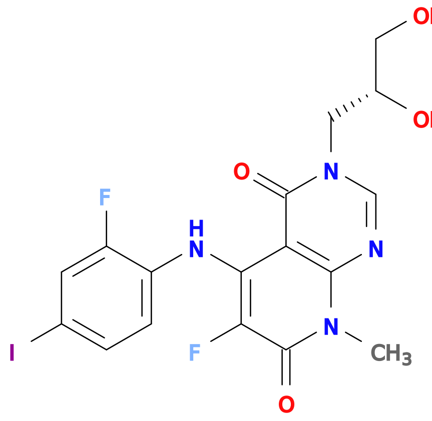

Database identifiers

ChEMBLCompound	CHEMBL1615025
ZINC	ZINC43196550
eMolecules	36516879

Ranking

	Rank	Score
Drosophila	204/697	0.697
C. elegans	277/591	0.203

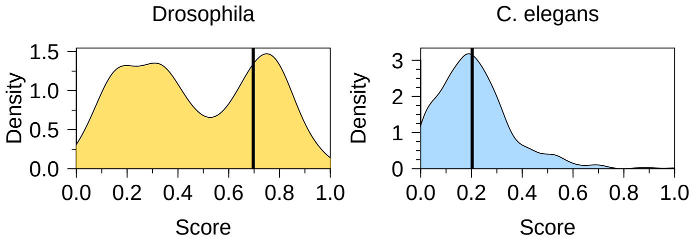

	Ageing implication	Domain conservation	Binding site conservation	Binding affinity	Bioavailability	Lipinski	Promiscuity	Purchasability	Drug approval	**Total**
Drosophila	0.81	0.965	1.0	0.92	(0.9)	-0.05	-0.0	0.1	0.0	0.697
C. elegans	0.81	0.94	0.983	0.92	0.223	-0.05	-0.0	0.1	0.0	0.203

Names

No synonyms found

Roles

ChEBI entry None has no roles

Status

Approved drug (according to ChEMBL)	No
Number of Rule of 5 violations	1
Binding affinity to original target in log units (RF-Score prediction)	7.44
Burns *C. elegans* bioavailability prediction	-3.96

Compound Target Characteristics

Dual specificity mitogen-activated protein kinase kinase 1

Best gene implication in ageing for this target family came from gene Q02750 annotated in UniProt release 2014_02. Annotation GO subterm of 7568 (aging) was Inferred from Mutant Phenotype

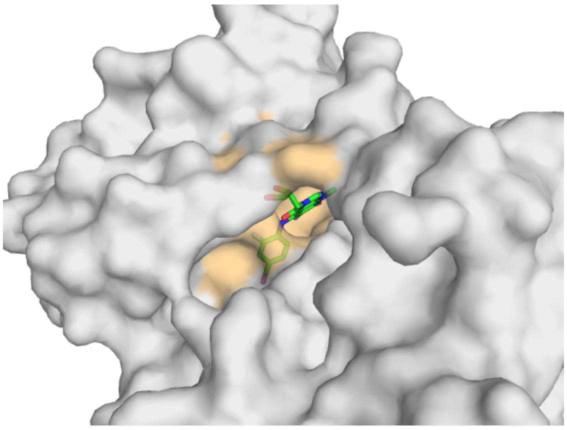

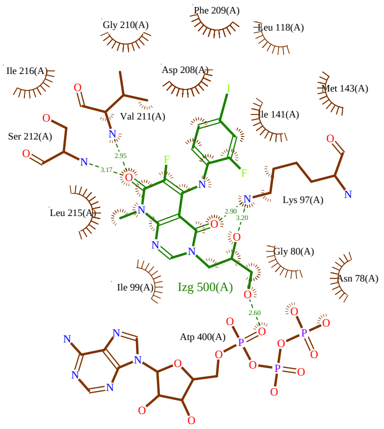

protein	amino acids contacts (binding site)														
PDB:3pp1:chainA:Q02750	N	G	K	I	L	L	V	I	M	D	F	G	V	S	L	I
sp:Q02750:MP2K1_HUMAN	N	G	K	I	L	L	V	I	M	D	F	G	V	S	L	I
tr:A4QPA9:A4QPA9_HUMAN	N	G	K	I	L	L	V	I	M	D	F	G	V	S	L	I
sp:Q01986:MP2K1_RAT	N	G	K	I	L	L	V	I	M	D	F	G	V	S	L	I
tr:Q3TMJ8:Q3TMJ8_MOUSE	N	G	K	I	L	L	V	I	M	D	F	G	V	S	L	I
sp:P31938:MP2K1_MOUSE	N	G	K	I	L	L	V	I	M	D	F	G	V	S	L	I
sp:Q24324:DSOR1_DROME	N	G	K	I	L	L	V	I	M	D	F	G	V	S	L	I
sp:Q10664:MEK2_CAEEL	N	G	K	V	L	L	V	I	M	D	F	G	V	S	L	I
sp:P08018:PBS2_YEAST	N	G	K	V	L	L	V	M	M	D	F	G	V	S	L	V

protein	whole protein ident	whole protein simil	domain-based ident	domain-based simil	contact-based ident	contact-based simil
PDB:3pp1:chainA:Q02750	1.0	1.0	1.0	1.0	1.0	1.0
sp:Q02750:MP2K1_HUMAN	1.0	1.0	1.0	1.0	1.0	1.0
tr:A4QPA9:A4QPA9_HUMAN	1.0	1.0	1.0	1.0	1.0	1.0
sp:Q01986:MP2K1_RAT	0.99	1.0	1.0	1.0	1.0	1.0
tr:Q3TMJ8:Q3TMJ8_MOUSE	0.99	1.0	1.0	1.0	1.0	1.0
sp:P31938:MP2K1_MOUSE	0.99	1.0	1.0	1.0	1.0	1.0
sp:Q24324:DSOR1_DROME	0.6	0.82	0.75	0.93	1.0	1.0
sp:Q10664:MEK2_CAEEL	0.48	0.74	0.62	0.88	0.94	0.98
sp:P08018:PBS2_YEAST	0.2	0.36	0.45	0.74	0.81	0.96

Dsor1 (FBgn0010269) associated phenotypes

dominant, lethal - all die before end of pupal stage, neuroanatomy defective, polyphasic, some die during immature adult stage, some die during pupal stage

(Information from FlyBase)

Dsor1 (UniProt:Q24324) annotation

Function: Required downstream of Raf in the sevenless (sev), torso (tor), and Drosophila EGF receptor homolog (DER) signal transduction pathways. Involved in both positive regulation (at the posterior terminus) and negative regulation (at the anterior domain) of tll, as in other terminal class gene products, maybe via the ERK-A kinase. (PubMed:8381718).
Developmental stage: Expressed both maternally and zygotically. (PubMed:8381718).
Ptm: Phosphorylation on Ser/Thr by MAP kinase kinase kinases regulates positively the kinase activity.

(Information from UniProt)

mek-2 (WBGene00003186) associated phenotypes
AWA odorant chemotaxis defective, AWC odorant chemotaxis defective, bacterially unswollen, constipated, germ cell arrest, germ nuclei rachis, lethal, metabolic pathway variant, no oocytes, pachytene region organization variant, pathogen susceptibility increased, rod like larval lethal, sterile, vulval cell lineage variant, vulvaless

(Information from WormBase)

mek-2 (UniProt:Q10664) annotation
Function: Functions in the let-60 Ras signaling pathway; acts downstream of lin-45 raf kinase, but before the sur-1/mpK-1 gene product in controlling vulval cell differentiation.
Enzyme regulation: Activated by tyrosine and threoninephosphorylation catalyzed by MAP kinase kinase kinases.

(Information from UniProt)
